# Involuntary protection against dermatosis: A preliminary observation on trypophobia

**DOI:** 10.1186/s13104-017-2953-6

**Published:** 2017-12-01

**Authors:** Yuki Yamada, Kyoshiro Sasaki

**Affiliations:** 1 Faculty of Arts and Science, Kyushu University, 744 Motooka, Nishi-ku, Fukuoka, 819-0395 Japan; 2 Faculty of Science and Engineering, Waseda University, 3-4-1 Ohkubo, Shinjuku-ku, Tokyo, 169-8555 Japan; 3 Japan Society for the Promotion of Science, 5-3-1 Kojimachi, Chiyoda-ku, Tokyo, 102-0083 Japan

**Keywords:** Cognition, Emotion, Embodiment, Disgust, Discomfort

## Abstract

**Objective:**

Trypophobia refers to the intense negative emotions evoked by exposure to repeated visual patterns like a honeycomb. We propose a cognitive mechanism that can explain why such negative emotions are triggered by trypophobic objects, primarily through automatic and involuntary avoidance of skin diseases, which is also called as the Involuntary Protection Against Dermatosis (IPAD) hypothesis.

**Results:**

We asked 856 participants to evaluate the discomfort evoked by trypophobic images and to report their past and current skin-related medical problems. Results showed that participants with a history of skin problems rated the pictures as evoking high discomfort as compared to those without skin problems. We conducted another survey to replicate the original survey using additional 690 participants, which confirmed the reliability of the current findings. The current study presents preliminary observational data that supports the IPAD hypothesis and suggests ways to reduce maladaptive emotional reactions toward trypophobic objects.

## Introduction

Repeated visual patterns (e.g., a honeycomb) sometimes evoke intensely negative emotional reactions, insomuch that the reactions appear to be a kind of pathological problem (trypophobia). Images that induce trypophobia (trypophobic images) are perceived as entertainment. A search of the word “trypophobia” on any internet search engine reveals that many people enjoy viewing such images. There are also many Facebook groups associated with “trypophobia,” which indicates the increasing public interest in trypophobia. On the other hand, as with other phobias, trypophobia also causes maladaptation in daily life. For example, acute body reactions such as vomiting or muscle jerking are induced when some people see trypophobic images. Effective intervention for these symptoms has not been found yet; therefore, the present study aimed to clarify the internal mechanism of trypophobia and to provide basic knowledge for the development of interventions for it.

Scientific arguments regarding trypophobia have been proposed only since 2013 [1]. Therefore, researchers have not reached a consensus on an explanation for this phenomenon. Two explanations are commonly discussed and preferred among researchers. One focuses on the body surface pattern of harmful animals. Researchers explain that observers experience discomfort from trypophobic objects in order to rapidly avoid them because most poisonous animals have a similar pattern on their surface [1]. Another explanation is based on the notion that patterns of trypophobic objects are similar to scars and sores [2, 3]. Such visual information is associated with animal reminder disgust, which is a subclass of the emotion of disgust, and is linked with primitive fears of animals, including humans, regarding death and body damage [4].

The first “poisonous animal avoidance” hypothesis cannot explain why observers sometimes experience negative emotions from harmless objects such as a lotus seedpod. A detector of poisonous animals might set its threshold lower to successfully detect a minute danger, but an extremely low threshold should make the detector useless. This detection of various kinds of harmless repeated patterns that cause intense negative emotions as dangerous seems unnecessary from the perspective of adapting to natural environments [5]. The second “animal reminder disgust” hypothesis also cannot explain why the lotus seedpod induces animal reminder disgust, as it is not related to death nor body damage.

In this paper, we propose a new possible explanation, the “involuntary protection against dermatosis” (IPAD) hypothesis. This is a hybrid version of the two previous hypotheses. According to the IPAD hypothesis, observers experience negative emotions on being exposed to trypophobic objects so that they can involuntarily avoid them because contagious skin diseases have a common visual pattern that is similar to that observed in trypophobic objects. This notion is supported by empirical data indicating that trypophobia is related to core disgust (i.e., emotional avoidance of pathogen infection) [6, 7]. Briefly, based on the IPAD hypothesis, we experience negative emotions from trypophobic objects because their surface is associated with skin diseases, and thus, the avoidance reaction to the pathogen occurs. The role of this skin pathogen avoidance has been discussed recently by two studies [5, 8]. Specifically, Sasaki et al. [5] discussed the neural mechanism of trypophobia, in which the early processing of visual features, such as image statistics, automatically and involuntarily activated the amygdala. This finding suggests that the IPAD hypothesis involves involuntariness of emotional reactions. Kupfer and Le [8] presented evidence for the correlational relationship between trypophobia and the core disgust found by Imaizumi et al. [6]. Moreover, they reported that some of their observers mentioned “skin” in the introspective descriptions. While the causal relationship between trypophobia and skin diseases is still unclear, these findings support the IPAD hypothesis.

The aim of the present study was to provide preliminary data to test the IPAD hypothesis. Thus, we used an online survey to present trypophobic images to people who have ever suffered from skin diseases and those who have not, and asked them to assess the discomfort experienced in response to the images. The IPAD hypothesis predicted that people who had suffered from skin diseases would be more defensive against infection, and therefore, they would be more uncomfortable with trypophobic images as compared to those who had never suffered from skin diseases. The other previous hypotheses mentioned above cannot provide this prediction. In addition, in order to guarantee the reliability of our findings, we replicated the original survey using another group of participants.

## Main text

### Surveys

For the preliminary observation, 1000 people were recruited online through Yahoo! Crowdsourcing, but only 856 participants (494 males and 362 females; Mean age = 41.5 years, SD = 10.0 years) completed the survey. The purpose of the study was not revealed to the participants. Using G*Power 3.1, we calculated the sample size to have sufficient power to detect a relatively small effect, Cohen’s *d* = .30. In this study, we did not focus on university students, who are conventionally used in psychological studies, but rather selected a more general population. Therefore, we did not specifically set criteria for the recruitment of participants. In the survey, the participants were asked to rate their discomfort in response to 10 trypophobic images used in previous studies [5, 9], using an 11-point scale (0: no discomfort at all; 10: extreme discomfort). The following instruction was provided to the participants: “Please rate the discomfort experienced on seeing the image.” The participants also reported their past and current skin-related medical problems in response to the question “Even if it was mildly symptomatic, please tell us if you have any history of skin diseases or currently suffer from any skin disease.” If more than one history was reported, the participants were classified in the “with history group,” otherwise they were classified in the “without history group.” For all analyses, we set the significance level at α = .05.

**Fig. 1:**
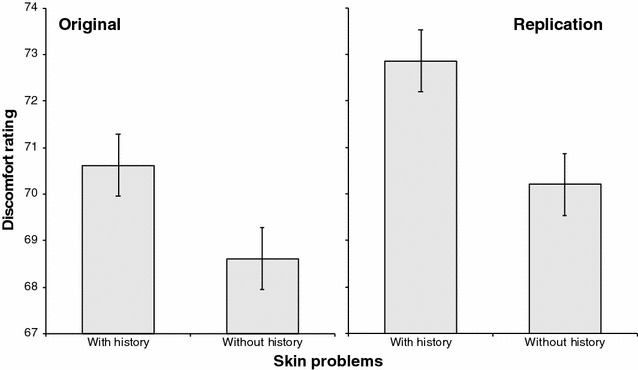
The results of the original (left panel) and replicated (right panel) surveys. Error bars represent standard errors of the means

We first analyzed the discomfort ratings using the Mann–Whitney U test (unless otherwise noted, the p values presented here are two-sided), because the Shapiro–Wilk test suggested that the data significantly deviated from normality (*p* = .001) and the Levene test suggested that variances did not significantly differ between the groups (*p* = .799). As a result, as shown in Fig. 1, participants with a history of skin problems reported experiencing significantly higher discomfort than did those without skin problems (*p* = .048). Moreover, we compared the mean age between the groups. Table 1 shows data on the age and gender of the groups with and without skin disease history. A two-sample *t* test (Shapiro–Wilk test: *p* = .242; Levene test: *p* = .811) suggested no difference in age between the groups (*t*(853) = 1.818, *p* = .069). A Chi squared test suggested a significant difference in the male/female ratio between the groups (χ^2^(1) = 10.127, *p* = .001, Cramér’s *V* = .109).

**Table 1:** The summary of the groups in the original survey

	Age	Gender
With history	42.421 (SD = 9.920)	M = 135, F = 136
Without history	41.092 (SD = 9.950)	M = 359, F = 226

*M* number of male participants, *F* number of female participants

We further preformed a confirmatory survey using another group of participants. This time, 690 out of 753 participants (328 males and 362 females; Mean age = 39.6 years, SD = 9.9 years) completed the survey. The Shapiro–Wilk test suggested that the data significantly deviated from normality (*p* = .001) and the Levene test suggested that the variances did not significantly differ between the groups (*p* = .303). As shown in Fig. 1, the Mann–Whitney U test showed that participants with a history of skin problems reported experiencing significantly higher discomfort than did those without skin problems (*p* = .044). Table 2 shows the data in the age and gender of the groups with and without history of skin diseases in this confirmatory survey. A two-sample *t*-test (Shapiro–Wilk test: *p* = .053; Levene test: *p* = .776) suggested no differences in age between the groups (*t*(685) = .147, *p* = .883). A Chi squared test suggested a significant difference in the male/female ratio between the groups (χ^2^(1) = 4.76, *p* = .029, Cramér’s *V* = .083).

**Table 2:** The summary of the groups in the replicated survey

	Age	Gender
With history	39.662 (SD = 9.950)	M = 94, F = 132
Without history	39.543 (SD = 9.907)	M = 234, F = 230

*M* number of male participants, *F* number of female participants

The findings of the original survey and its replication together suggest that the history of skin problems is related to trypophobic discomfort. This evidence reliably supports the IPAD hypothesis. According to this hypothesis, the present results suggest the presence of a cognitive processing mechanism of dermatosis-avoidance. That is, people who have suffered skin problems present a learned avoidance against trypophobic objects that can be a potential source of pathogen infection that may cause dermatosis. However, the male/female ratio was different between the groups in both the surveys. Although the ratios were inconsistent between the two surveys (i.e., there were more male than female participants in the group without skin history in the original survey and more female than male participants in the group with skin history in the replication survey), the possibility remains that this difference contaminated the difference observed in trypophobic discomfort difference. However, it is still unclear what kind of contamination this factor may have caused.

## Limitations

In the present paper, we newly proposed the IPAD hypothesis and conducted two surveys that provided supportive evidence for this hypothesis. The findings of the current study have significance in presenting quantitative data supporting the qualitative data in the open-ended responses presented by a previous study [8]. However, the present surveys could not reveal a clear causal relationship between trypophobia and skin diseases. For example, it is necessary to verify the effect of skin diseases while controlling for gender differences. Although the effect of gender differences on trypophobic discomfort becomes an interesting research question in future research, note that previous studies using the trypophobia scale have not reported gender differences [5, 6, 8–10]. The present survey should be considered a preliminary one. To rigorously examine the most important point regarding causal relations, we further propose that researchers conduct psychological experiments in which the observers’ skin protection conditions are manipulated. The first could be an experiment in which an experimenter manipulates protected skin areas by changing the length of the sleeves of the observers’ clothes. The second could be an experiment in which an experimenter manipulates pharmacological inhibition by applying antibiotic or antiseptic agents or skin protectants on the skin. In both the experiments, the IPAD hypothesis predicts that the treated observers would show less negative emotional reactions toward the trypophobic objects than those without any treatments (i.e., a control group). This is because a trypophobia-related emotional system in the treated observers would lead them to estimate the potential risk of the trypophobic objects as lower due to the exogenously increased protection condition. On the other hand, the previous two hypotheses do not predict this pattern of results. Therefore, these experiments would distinguish the previous hypotheses from our IPAD hypothesis.

Moreover, the neural mechanism of trypophobia needs to be specified. Previous studies have repeatedly presented the possibility that trypophobia is associated with core disgust [6–8] and behavioral immune system [5]. These discussions presume that human beings have an internal system that attempts to keep their bodies away from pathogens. Trypophobia, a strong negative reaction to mere repetitive visual patterns, can be said to be the result of an excessive response of this system [8]. However, as with what triggers the response of the internal system (it may be IPAD), it is also unclear what the mechanism of the system is. Regarding the neural basis related to this system, Sasaki et al. [5] suggested the importance of the amygdala-based processing that operates automatically and involuntarily. They also indicated that visual information at middle to low spatial frequencies is sent to the amygdala through a colliculo-pulvinar pathway [11, 12]. Further experimentation on this point will lead to a deeper understanding of the psychological and neural mechanisms of trypophobia.

The empirical assessment of the IPAD hypothesis through the proposed experiments is really important because only the IPAD hypothesis, rather than the previous hypotheses, can help suggest ways to reduce trypophobia. As we mentioned earlier, trypophobia sometimes causes severe pathological problems, and hence, effective interventions are needed. If the IPAD hypothesis is valid, then the symptomatic negative reactions of the people experiencing difficulty on seeing repetitive visual patterns in everyday life could be easily alleviated through physical and pharmacological interventions. The present findings reveal the possibility that it is important to avoid infections or skin diseases as much as possible in order to reduce the trypophobic discomfort evoked by such visual patterns. However, its applicability is low. Therefore, there is an urgent need to directly examine whether trypophobic discomfort decreases by protecting the skin.

## Abbreviation

IPAD: involuntary protection against dermatosis
